# Health-related quality of life and its related factors in coronary heart disease patients: results from the Henan Rural Cohort study

**DOI:** 10.1038/s41598-021-84554-6

**Published:** 2021-03-03

**Authors:** Yong-xia Mei, Hui Wu, Huan-yun Zhang, Jian Hou, Zhen-xiang Zhang, Wei Liao, Xiao-tian Liu, Sheng-xiang Sang, Zhen-xing Mao, Dong-bin Yang, Chong-jian Wang, Wei-hong Zhang

**Affiliations:** 1 School of Nursing and Health, Zhengzhou University, No. 100 Kexue Road, Zhengzhou, 450001 People’s Republic of China; 2 Department of Epidemiology and Biostatistics, College of Public Health, Zhengzhou University, 100 Kexue Avenue, Zhengzhou, 450001 Henan People’s Republic of China; 3 The People’s Hospital of Hebi, 115 Jiuzhou Rd, Hebi, 458000 Henan People’s Republic of China; 4 School of Public Health, Xinxiang Medical University, 601 Jinsui Rd, Xinxiang, 453000 Henan People’s Republic of China; 5 School of Public Health, Tianjin Medical University, 22 Qixiangtai Rd, Heping District, Tianjin, 300070 People’s Republic of China; 6 Henan Province General Medical Educations and Research Center, Xinxiang, People’s Republic of China; 7 Department of Neurosurgery of Hebi People’s Hospital, Hebi Neuroanatomical Laboratory, Hebi, 458030 People’s Republic of China

**Keywords:** Cardiology, Health care, Risk factors

## Abstract

The aims were to identify the possible influencing factors of health-related quality of life (HRQoL) and its domain-specific scores in patients with coronary heart disease (CHD). A total of 1247 patients with CHD from the Henan Rural Cohort Study (n = 39,259) were included in this study. The Chinese version of the European Quality of Life Five Dimension Five level scale (EQ-5D-5L) and Visual Analogue Scale (VAS) were used to evaluate HRQoL in patients with CHD. Tobit regression, generalized linear models and binary logistic regression were applied to determine the potential factors influencing the EQ-5D utility, as well as each domain, and the VAS. CHD patients had lower per capita monthly actual income, and higher rates of diabetes mellitus, stroke, anxiety and poor sleep quality, which significantly decreased EQ-5D index and VAS scores. In addition, sex, older age, education, not having a spouse, ever drinking alcohol, a high-fat diet, physical activity, hypertension and depression affected the various domain-specific EQ-5D scores in CHD patients. CHD patients in rural areas have a lower HRQoL. Factors associated with the EQ-5D index, including each domain, and the VAS need attention. CHD patients in rural areas need to be managed systematically.

## Introduction

Coronary heart disease (CHD) is still a major and costly health problem worldwide^1^. According to a report from the European Society of Cardiology (ESC) in 2019, there are international inequalities in disease burden and health care delivery across the 56 member countries, and cardiovascular disease mortality was higher in middle-income countries due to being severely under-resourced^2^. In China, ischaemic heart disease was one of the leading causes of death and disability adjusted of life years (DALYs) at the national level in 2017^3^, and the mortality of CHD in rural areas was higher than that in urban areas^4^. Moreover, the prevalence of cardiovascular disease (CVD) is still on the rise, and there are 11 million CHD patients^5^.

Health-related quality of life (HRQoL) has been clearly shown to affect health, and includes perceptions of both physical and mental health and their correlates on the individual level, according to the National Center for Chronic Disease Prevention and Health Promotion^6^. HRQoL is an important outcome in patients with CHD; however adults with CHD reported reduced HRQoL^7^. Moreover, CHD patients with lower HRQoL had a higher risk of composite CHD/cerebrovascular outcomes^8^. Identification of influencing factors related to HRQoL can provide more effective strategies to improve the HRQoL and outcomes of patients with CHD.

Previous research has focused on HRQoL for a long time; however, the level of HRQoL and its predicators vary due to cultural differences and the use of different measurement tools^9,10^. The European Quality of Life Five Dimensions (EQ-5D) instrument is a frequently used generic preference-based tool to assess the HRQoL of various patients, and the EQ-5D-5 level scale (EQ-5D-5L) have obvious advantages that can reduce the ceiling effect compared with the EQ-5D-3 level tool^11–13^. However, most researchers have only focused on the influencing factors of the EQ-5D index, and they have paid little attention to the influencing factors of each domain. Due to the multidimensional structure of HRQoL, understanding the influencing factors of each domain of the EQ-5D-5L will provide more detailed insight into the problems that CHD patients suffer from^9^. A previous study assessed the differences between CHD patient groups from 24 European countries in terms of the problems reported on the EQ-5D scale; the comparison across countries showed major differences^10^.

Henan is a populous province located in central China, with a rural population accounting for half of the total population^4^. Rural people had have worse HRQoL than people living in rural urban areas^14^. Moreover, the CVD burden in Henan Province appeared to be higher than those in coastal provinces with higher economic development^15^. Data regarding the overall and domain-specific HRQoL of CHD patients in rural areas in Henan remain limited, and further study on the HRQoL of patients with CHD in the Asian context is needed^16^. To provide effective and useful interventions to improve the HRQoL of urban patients with CHD, this study aimed to evaluate the overall and domain-specific HRQoL of urban CHD patients and their possible determinants within a large sample size using the Chinese EQ-5D-5L cross-walk value sets.

## Methods

### Sample

The participants were from the Henan Rural Cohort study, which was registered in the Chinese Clinical Trial Register (Registration Number: ChiCTR-OOC-15006699, http://www.chictr.org.cn/showproj.aspx?proj=11375^17^. This baseline study was carried out in five rural regions (south, central, north, east and west) of Henan Province in China between July 2015 and September 2017 in adults aged 18–79 years. This study was approved by the Zhengzhou University Life Science Ethics Committee and written informed consent was obtained from all participants (Ethic approval code: [2015] MEC (S128)). In total, 39,259 people responded to the survey, and 4.4% of them (1734) reported having CHD. A detailed description of the survey in the Henan rural cohort has been previously published^17^. Data from the respondents who reported having CHD elicited, and incomplete EQ-5D-5L information was removed; thus, the final dataset in this study contained data for 1247 CHD patients.

The sociodemographic characteristics and clinical condition of the enrolled patients with CHD are shown in Table 2. There were 1247 participants in total. The mean(SD) age for the analysis sample was 61.73 years (8.62), ranging from 25 to 79 years old; 64.1% were female, 88.1% had a spouse, 43.5% had a junior high school or above education, and 41.6% had a per capita monthly actual income lower than 72 dollars. In this study, 73.60% of participants never smoked, 80.30% of participants never drink, 88.70% of participants did not have a high-fat diet, and 56.90% did not have a vegetable and fruit diet. A total of 36.90% of the participants reported moderate-intensity physical activity. In this study, 26.70% of participants had an abnormal waist-to-hip ratio, 31.4% had a normal BMI, and 59.60% centripetal obesity. A total of 35.5% of participants had hypertension, 13.2% had diabetes mellitus and 16.7% had stroke. A total of 9.60% participants had anxiety, 10.8% had depression, and 34.3% had poor sleep quality.

### Measurements and definitions

A standardized questionnaire was administered by trained research staff through face-to-face interviews to collect detailed information on sociodemographic variables (e.g., age, sex, marital status, education and personal income, etc.) and lifestyle variables (e.g., smoking, alcohol consumption, diet, etc.), details are shown in Table 1. Moreover, physical activity intensity over the last 7 days was measured by International Physical Activity Questionnaires (short form) and categorized as mild, moderate or intense^18^. Body mass index (BMI) was categorized as low (< 18.5 kg/m^2^), normal (≥ 18.5 kg/m^2^ and < 24.0 kg/m^2^), overweight (≥ 24.0 kg/m^2^ and < 28.0 kg/m^2^) and obese (≥ 28.0 kg/m^2^) BMI^19^; only 1244 participants had complete BMI data. Centripetal obesity was defined as a waist circumference (WC) was ≥ 90 cm in males or ≥ 80 cm in females^19^, and was categorized as yes or no; only 1245 participants had WC data. The normal waist-to-hip ratio was defined as a ratio ≥ 0.9 in a male CHD patient and ≥ 0.8 in females^20^; and only 1244 participants had complete data for this variable.

**Table 1:** Definitions and coding schema for patients’ sociodemographic variables.

Variables	Definitions and coding schema
Age	< 55 = 1; 55- = 2; ≥ 65 = 3
Sex	Male = 1; Female = 2
Marital status	Was categorized as married/cohabiting and widowed/unmarried/divorce/separation married/cohabiting = 1; widowed/unmarried/divorce/separation = 2
Spouse	Was defined as whether a CHD patient was accompanied by a spouse, and was categorized as yes or no No = 0; Yes = 1
Educational level	Was categorized as illiterate, primary school, junior high school or above illiterate = 1; primary school = 2; junior high school and above = 3
Per capita monthly actual income	Was categorized as < 72$ (US dollars), 72 ~ 143$, and 143 ~ $ < 72 = 1; 72- = 2; ≥ 143 ~ = 3
Smoking status	Was defined as at least one cigarette per day for previous six months Never = 0; Current = 1; Former = 3
Drinking status	Was defined as 12 times alcohol consumption per year Never = 0; Current = 1; Former = 3
High-fat diet	Was defined as taking at least 75 g fat per day No = 0; Yes = 1
Vegetable and fruit diet	Was defined as taking at least 500 g vegetable or fruit per day No = 0; Yes = 1

Hypertension was defined as whether a male CHD patient’s blood pressure was ≥ 140/90 mmHg, or self-reported hypertension was diagnosed by a physician and current use of antihypertensive medicines was reported^21^; this variable was categorized as yes or no. Diabetes mellitus was defined as fasting blood glucose ≥ 7.0 mmol/L, or by self-reported diagnosis of a doctor and current use of hypoglycaemic agents; this variables was categorized as yes or no. Stroke was defined as ever having been hospitalized for stroke and was categorized as yes or no. Participants wore light clothing when anthropometric variables were measured, and their body weight and height were measured twice to the nearest 0.1 kg and 0.1 cm, respectively. In addition, blood pressure was measured using an electronic sphygmomanometer (HEM-770A Fuzzy, Omron, Japan) in a seated position after 5 min of rest, and fasting blood glucose was measured by using venous blood samples after overnight fasting.

Anxiety over the last 14 days was assessed by the 2-item generalized anxiety disorder scale (GAD-2), and a GAD-2 score greater than or equal to 3 indicated anxiety^22^. Depression over the last 14 days was assessed by the 2-item Patient Health Questionnaire-2 (PHQ-2), and a PHQ-2 score greater than or equal to 2 indicated depression^23^. A total of 1246 participants completed the anxiety and depression assessment. Sleep quality was assessed by the Pittsburgh Sleep Quality Index (PSQI) over the previous 30 days, and a PSQI score greater than or equal to 5 indicated poor sleep quality^24^. A total of 1229 CHD patients completed the sleep quality assessment.

HRQoL was measured using the Chinese EQ-5D-5L, which is a generic preference-based instrument that consisted of a descriptive system and the EQ Visual Analogue Scale (EQ-VAS)^25^. Mobility (MO), self-care (SC), usual activities (UA), pain/discomfort (PD) and anxiety/depression (AD) were the domains included in the descriptive system, and each was scored from 1 (no problem) to 5 (extreme problems)^26^. A total of 3125 possible health states with a combination of answers from 11,111 (full health) to 55,555 (worst health), were represented by a single EQ-5D-5L utility index from − 0.391 (for 55,555) to 1.000 for (11,111) using the Chinese cross-walk value set^27^. Participants were asked to evaluate their health state, which they reported on a vertical thermometer-like scale ranging from 0 (worst imaginable health) to 100 (best imaginable health) on the EQ-VAS^26^. More detailed information on the cohort has been described elsewhere^17^.

### Data analysis

Data were analysed using STATA 15 for Windows and IBM SPSS statistics software package, version 21.0 for windows (Chicago, IL, USA). Means and standard deviations (SDs) were used to describe the continuous variable data, and frequencies and percentages were used to describe the categorical variable data. The association between the EQ-5D-5L utility index and VAS scores was assessed by the Spearman correlation test. Univariate analysis of the associations between the EQ-5D domains and the various participants’ characteristics (age, sex, education, spouse/single, per capita monthly actual income, smoking status, drinking status, high-fat diet, vegetable and fruit diet, physical activity intensity, BMI, centripetal obesity, waist-to-hip ratio, hypertension, diabetes mellitus, stroke, anxiety, depression, sleep quality) were tested by the chi square test. Binary logistic regressions were used to assess the potential influencing factors (those that had significant differences by chi square test; i.e., all the various participants characteristics except vegetable and fruit diet and diabetes mellitus) of the EQ-5D domain scores. Associations between all the various participants characteristics and the EQ-5D utility index and VAS score were tested by Mann–Whitney *U* test (two groups) and Kruskal–Wallis one-way analysis of variance (multiple groups) due to the abnormal distribution of the EQ-5D utility index and VAS score. A Tobit regression model was applied to explore the potential influencing factors of the EQ-5D index score (those that had significant associations with the EQ-5D utility index or VAS score; i.e., all participants characteristics except centripetal obesity); those methods was chosen because the EQ-5D utility data were skewed and the utility score was censored at 1^28^. A generalized linear model (GLM) was used to examine the potential influencing factors (same factors as the Tobit regression model) of EQ-5D VAS scores; those methods were chosen because the VAS scores data were not normally distributed. Statistical significance was set at *P* ≤ 0.05 in all analyses.

### Guidelines and regulations statement

We confirm that all methods in our manuscript were carried out in accordance with relevant guidelines and regulations.

## Results

### Health problems reported

The PD dimension of the EQ-5D-5L was the most frequently reported problem (34.9%), followed by the MO (21.8%), UA (12.6%) and AD (12.5%) dimensions. Problems with SC were the least reported (8.0%). The frequencies of problems with PD, MO, UA, AD and SC were all significantly different among those participants who reported stroke, anxiety, depression, poor sleep quality and drinking alcohol (Table 2). In addition, the frequencies of different problems were significantly different among participants with various conditions, as shown in Table 2.

**Table 2:** Prevalence of having problems in each EQ-5D dimension among patients with CHD (n = 1247).

Subject characteristics	n (%)	MO	SC	UA	PD	AD	Mean utility index (SD)	Mean VAS scores (SD)
**Age (years)**								
< 55	275 (22.1)	39 (14.2)**	9 (3.3)**	21 (7.6)**	76 (27.6)**	27 (9.8)	0.95 (0.11)**	71.56 (17.87)**
55–65	461 (37.0)	80 (17.4)	24 (5.2)	41 (8.9)	141 (30.6)	56 (12.1)	0.93 (0.12)	71.13 (16.43)
65 ~	511 (41.0)	153 (29.9)	67 (13.1)	95 (18.6)	218 (42.7)	73 (14.3)	0.88 (0.21)	66.77 (18.00)
**Sex**								
Male	448 (35.90)	81 (18.1)*	28 (6.3)	42 (9.4)*	111 (24.8)**	38 (8.5)*	0.94 (0.13)**	69.95 (17.35)
Female	799 (64.10)	191 (23.9)	72 (9.0)	115 (14.4)	324 (40.6)	118(14.8)	0.90 (0.18)	69.15 (17.63)
**Education**								
Illiterate	309 (24.8)	82 (26.5)**	36 (11.7)**	56 (18.1)**	134 (43.4)**	45 (14.6)	0.88 (0.20)**	68.28 (18.87)*
Primary school	396 (31.8)	107 (27.0)	45 (11.4)	63 (15.9)	159 (40.2)	56 (14.1)	0.89 (0.18)	67.66 (17.78)
Other	542 (43.5)	83 (15.3)	19 (3.5)	38 (7.0)	142 (26.2)	55 (10.1)	0.95 (0.11)	71.39 (16.35)
**Spouse**								
Yes	1099 (88.1)	222 (20.2)**	83 (7.6)	124 (11.3)**	371 (33.8)*	131 (11.9)	0.92 (0.16)*	69.81 (17.38)*
No	148 (11.9)	50(33.8)	17 (11.5)	33 (22.3)	64 (43.2)	25 (16.9)	0.89 (0.16)	66.68 (18.43)
**Per capita monthly actual income ($)**								
< 72	519 (41.6)	149 (28.7)**	61 (11.8)**	80 (15.4)**	202 (38.9)*	74 (14.3)	0.89 (0.19)**	67.39 (18.06)**
72 ~ 143	393 (31.5)	70 (17.8)	27 (6.9)	55 (14.0)	128 (32.6)	47 (12.0)	0.92 (0.15)	70.01 (17.23)
143 ~	335 (26.9)	53 (15.8)	12 (3.6)	22 (6.6)	105 (31.3)	35 (10.4)	0.94 (0.13)	71.93 (16.70)
**Smoking status**								
Never	918 (73.60)	208 (22.7)	76 (8.3)	122 (13.3)	355 (38.7 )**	134 (14.6)*	0.90 (0.17)**	69.42 (17.56)
Former	168 (13.50)	32 (19.0)	12 (7.1)	19 (11.3)	44 (26.2)	11 (6.5)	0.94 (0.14)	67.70 (18.16)
Current	161 (12.90)	32 (19.9)	12 (7.5)	16 (9.9)	36 (22.4)	11 (6.8)	0.95 (0.12)	71.33 (16.57)
**Drinking status**								
Never	1001 (80.30)	233 (23.3)*	86 (8.6)*	135 (13.5)**	368 (36.8)*	137 (13.7)*	0.91 (0.17)**	68.82 (17.70)*
Former	110 (8.80)	26 (23.6)	11 (10.0)	19 (17.3)	32 (29.1)	7 (6.4)	0.92 (0.14)	69.13 (17.21)
Current	136 (10.90)	13 (9.6)	3 (2.2)	3 (2.2)	35 (34.9)	12 (8.8)	0.96 (0.07)	74.22 (15.85)
**High-fat diet**								
No	1106 (88.70)	257 (23.2)*	94 (8.5)	150 (13.6)*	393 (35.5)	143 (12.9 )	0.91 (0.17)*	69.00 (17.55)*
Yes	141 (11.30)	15 (10.6)	6 (4.3)	7 (5.0)	42 (29.8)	13 (9.2)	0.94 (0.11)	72.83 (17.04)
**Vegetable and fruit diet**								
No	709 (56.90)	156 (22.0)	55 (7.8)	97 (13.7)	241 (34.0_)	99 (14.0)	0.91 (0.17)	68.93 (17.84)
Yes	538 (43.10)	116 (21.6)	45 (8.4)	60 (11.2)	194 (36.1)	57 (10.6)	0.92 (0.16)	70.11 (17.11)
**Physical activity intensity**								
Mild	440 (35.30)	121 (27.5)*	51 (11.6)*	72 (16.4)*	142 (32.3)	57 (13.0)	0.89 (0.20)	67.04 (17.53)*
Moderate	460 (36.90)	83 (18.0)	29 (6.3)	43 (9.3)	167 (36.3)	57 (12.4)	0.93 (0.14)	70.74 (18.77)
Intense	374 (27.80)	68 (19.6)	20 (5.8)	42 (12.1)	126 (36.3)	42 (12.1)	0.92 (0.14)	70.74 (15.44)
**BMI (n = 1244)**								
< 18.5	26 (2.10)	8 (30.8)*	6 (23.1)*	6 (23.1)	8 (30.8)	6 (23.1)	0.85 (0.28)	60.19 (19.26 )*
18.5 ≤ BMI < 24.0	391 (31.4)	76 (19.4)	27 (6.9)	50 (12.8)	139 (35.5)	57 (14.6)	0.92 (0.15)	68.66 (17.11)
24.0 ≤ BMI < 28.0	523 (41.90)	101 (19.3)	37 (7.1)	56 (10.7)	167 (31.9)	59 (11.3)	0.92 (0.16)	70.80 (17.52)
≥ 28.0	304 (24.4)	86 (28.3)	29 (9.5)	44 (14.5)	119 (39.1)	33 (10.9)	0.90 (0.17)	68.98 (17.73)
**Centripetal obesity (n = 1245)**								
No	503 (40.40)	91 (18.1)*	35 (7.0)	57 (11.3)	167 (33.2)	66 (13.1)	0.92 (0.15)	68.98 (17.82)
Yes	742 (59.60)	180 (24.3)	64 (8.6)	99 (13.3)	267 (36.0)	89 (12.0)	0.91(0.17)	69.78 (17.34)
**Waist-to-hip ratio (n = 1244)**								
abnormal	332 (26.70)	212 (23.2)	79 (8.7)	120 (13.2)	332 (36.4)	109 (12.0)	0.93 (0.13)	69.66 (17.63)
Normal	912 (73.30)	59 (17.8)*	20 (6.0)	36 (10.8)	102 (30.7)	45 (13.6)	0.91 (0.17)	69.38 (17.52)
**Hypertension**								
No	804 (64.5)	154 (19.2 )*	55 (6.8)*	88 (10.9)*	277 (34.5)	97(12.1)	0.92 (0.16)	69.77 (17.65)
Yes	443 (35.5)	118 (26.6)	45 (10.2)	69 (15.6)	158 (35.7)	59 (13.3)	0.91 (0.17)	68.83 (17.31)
**Diabetes mellitus**								
No	1083 (86.8)	228 (21.1)	82 (7.6)	132 (12.2)	378 (34.9)	135 (12.5)	0.92 (0.14)	69.66 (17.38)
Yes	164 (13.2)	44 (26.8)	18 (11.0)	25 (15.2)	57 (34.8)	21 (12.8)	0.87 (0.25)	65.76 (18.14)
**Stroke**								
No	1039 (83.30)	212 (20.4)*	73 (7.0)*	117 (11.3)*	341 (32.8)*	115 (11.1)*	0.93 (0.14)**	71.12 (16.75)**
Yes	208 (16.70)	60 (28.8)	27 (13.0)	40 (19.2)	94 (45.2)	41 (19.7)	0.85 (0.25)	61.03 (18.91)
**Anxiety (n = 1246)**								
GAD-2 < 3	1127 (90.40)	236 (20.9)*	77 (6.8)**	127 (11.3)**	45 (37.9)**	98 (63.2)**	0.93 (0.14)**	70.54 (17.15)**
GAD-2 ≥ 3	119 (9.60)	35 (29.4)	23 (19.3)	29 (24.4)	74 (62.1)	57 (36.8)	0.76 (0.28)	58.87 (17.67)
**Depression (n = 1246)**								
PHQ < 2	1112 (89.20)	223 (20.1)**	70 (6.3)**	92 (68.7)**	350 (31.5)**	86 (7.7)**	0.93 (0.13)**	70.58 (16.98)**
PHQ ≥ 2	134 (10.80)	48 (35.8)	30 (22.4)	42 (31.3)	84 (62.7)	69 (51.5)	0.75 (0.28)	59.89 (19.15)
**Sleep quality (n = 1229)**								
PSQI ≤ 5	802 (64.5)	137 (17.1)**	39 (4.9)**	63 (7.9)**	223 (27.8)**	75 (9.4)**	0.94 (0.13)**	72.02 (16.72)**
PSQI > 5	427 (34.3)	130 (30.4)	58 (13.6)	90 (21.1)	206 (48.2)	79 (18.5)	0.86 (0.21)	64.85 (17.81)

*CHD* coronary heart disease, *MO* mobility, *SC* self-care, *UA* usual activities, *PD* pain/discomfort, *AD* anxiety/depression.

GAD-2 Generalized Anxiety Disorder Scale-2, PHQ-2 Patient Health Questionnaire-2, PSQI the Pittsburgh Sleep Quality Index.

*P < 0.05; **P < 0.001.

### EQ-5D-5L utility index and VAS scores

The mean utility index of people with CHD was 0.914 (SD, 0.164), ranging from -0.348 to 1.000 with a left-skewed distribution (skewness =  − 3.243). The 10 most common of the 3125 possible heath states represented the majority of the sample (82.4%). A state of 11,111 (no problem in any dimensions) was reported by 55.6% of the participants as shown in Table 3.

**Table 3:** The 10 most frequently reported EQ-5D-5L health states with mean utility scores and EQ-VAS values (n = 1247).

Health state	n	%	Cumulative %	Mean utility	Mean EQ-VAS (SD)
11,111	693	55.6	55.6	1.00	74.00 (15.28)
11,121	127	10.2	65.8	0.94	68.72 (18.87)
21,121	47	3.8	69.6	0.88	64.98 (15.60)
11,131	41	3.3	72.9	0.86	64.98 (15.50)
21,111	39	3.1	76.0	0.93	68.33 (18.19)
11,112	21	1.7	77.7	0.95	71.19 (10.48)
11,122	18	1.4	79.1	0.89	68.89 (17.11)
22,221	17	1.4	80.5	0.78	65.88 (15.33)
21,221	13	1.0	81.5	0.83	63.85 (15.57)
21,131	11	0.9	82.4	0.80	55.91 (20.84)

The mean VAS score of the participants was 69.44 (SD, 17.53), with a skewness of − 0.641. There was a moderate, positive statistically significant correlation between the utility index and VAS scores (r = 0.331, *P* < 0.001).

The EQ-5D-5L utility index and VAS scores stratified by different participant characteristics are presented in Table 2. The differences in the EQ-5D-5L utility index and VAS scores among different levels of age, education, spouse/single, per capita monthly actual income, alcohol consumption, high-fat diet, with stroke and anxiety, depression and poor sleep quality were statistically significant (*P* < 0.05) (Table 2).

### Factors associated with having problems in each EQ-5D dimension

**Figure 1:**
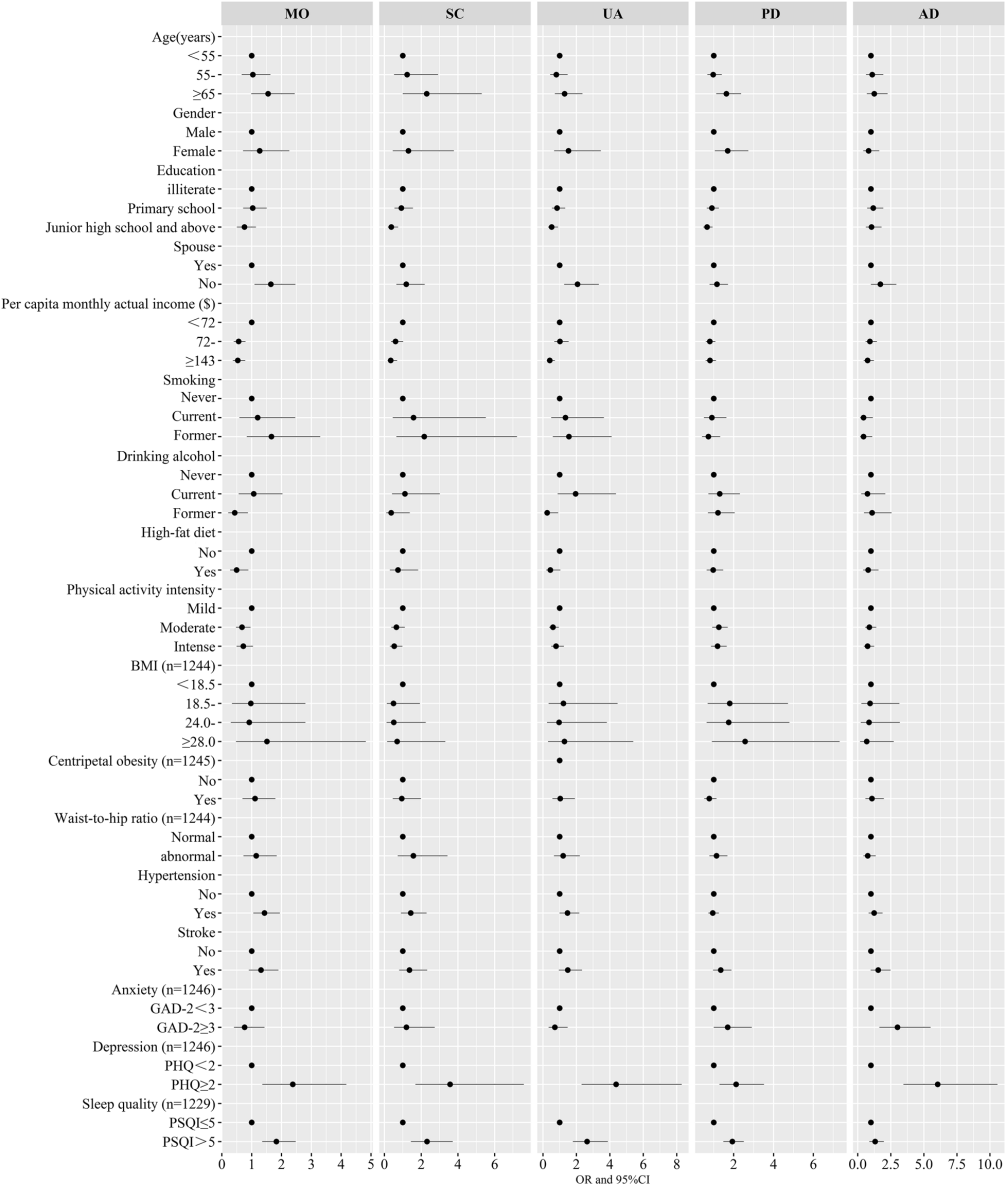
Risk factors for having problems in each EQ-5D dimension [Mobility (MO), Self-care (SC), Usual activities (UA), Pain/discomfort (PD) and Anxiety/depression (AD)] among patients with coronary heart disease (n = 1247) were analyzed by binary logistic regressions. The black dots with the corresponding error bars represented the estimated effect and 95% confidence intervals of the change in each of variables. A two-sided *P* value 0.05 was considered statistically significant. *Notes*: GAD-2 Generalized Anxiety Disorder Scale-2, PHQ-2 Patient Health Questionnaire-2, PSQI the Pittsburgh Sleep Quality Index.

The risk factors for having problems in each EQ-5D dimension, as determined by multiple logistic regression analysis, are shown in Fig. 1 and Table S1. Females and older CHD patients had an increased risk of suffering from PD [OR 1.7 (1.05–2.73) and 1.63 (1.12–2.37) respectively]. Higher education decreased the risk of having SC, UA, and PD problems. CHD patients without spouse had an increased risk of suffering from MO [OR 1.64 (1.10–2.47)] and UA [OR 2.07 (1.27–3.35)] problems. CHD patients with higher income had a decreased risk of MO, SC, and UA problems.

CHD patients who ever drank alcohol reported fewer MO [OR 0.44 (0.22–0.88)] and UA problems [OR 0.26 (0.07–0.92)] than those who never drank alcohol. CHD patients who consumed a high-fat diet had a decreased risk of MO problems [OR 0.50 (0.28–0.89)]. CHD patients who had a moderate physical activity level reported less MO [OR 0.68 (0.48–0.96)] and UA problem [OR 0.60 (0.38–0.95)], and those who had intense physical activity reported fewer SC [OR 0.54 (0.30–0.97)] problems than those who had mild physical activity. CHD patients with hypertension had an increased risk of suffering from problems with MO [OR 1.43 (1.05–1.95)].

CHD patients with anxiety were more likely to have problems with PD [OR 1.70 (1–2.91)] and AD [OR 3.00 (1.63–5.51)]. CHD patients with poor sleep quality had an increased risk of having problems with MO [OR 1.83 (1.35–2.48)], SC [OR 2.32 (1.44–3.72)], UA [OR 2.64 (1.79–3.88)], and PD [OR 1.93 (1.48–2.51)]. CHD patients with depression had an increased risk of having problems with MO [OR 2.38 (1.36–4.18)], SC [OR 3.58 (1.69–7.59)], UA [OR 4.38 (2.31–8.3)], PD [OR 2.12 (1.28–3.52)], and AD [OR 6.04 (3.45–10.57)].

### Factors associated with the EQ-5D-5L utility index and VAS scores

**Figure 2:**
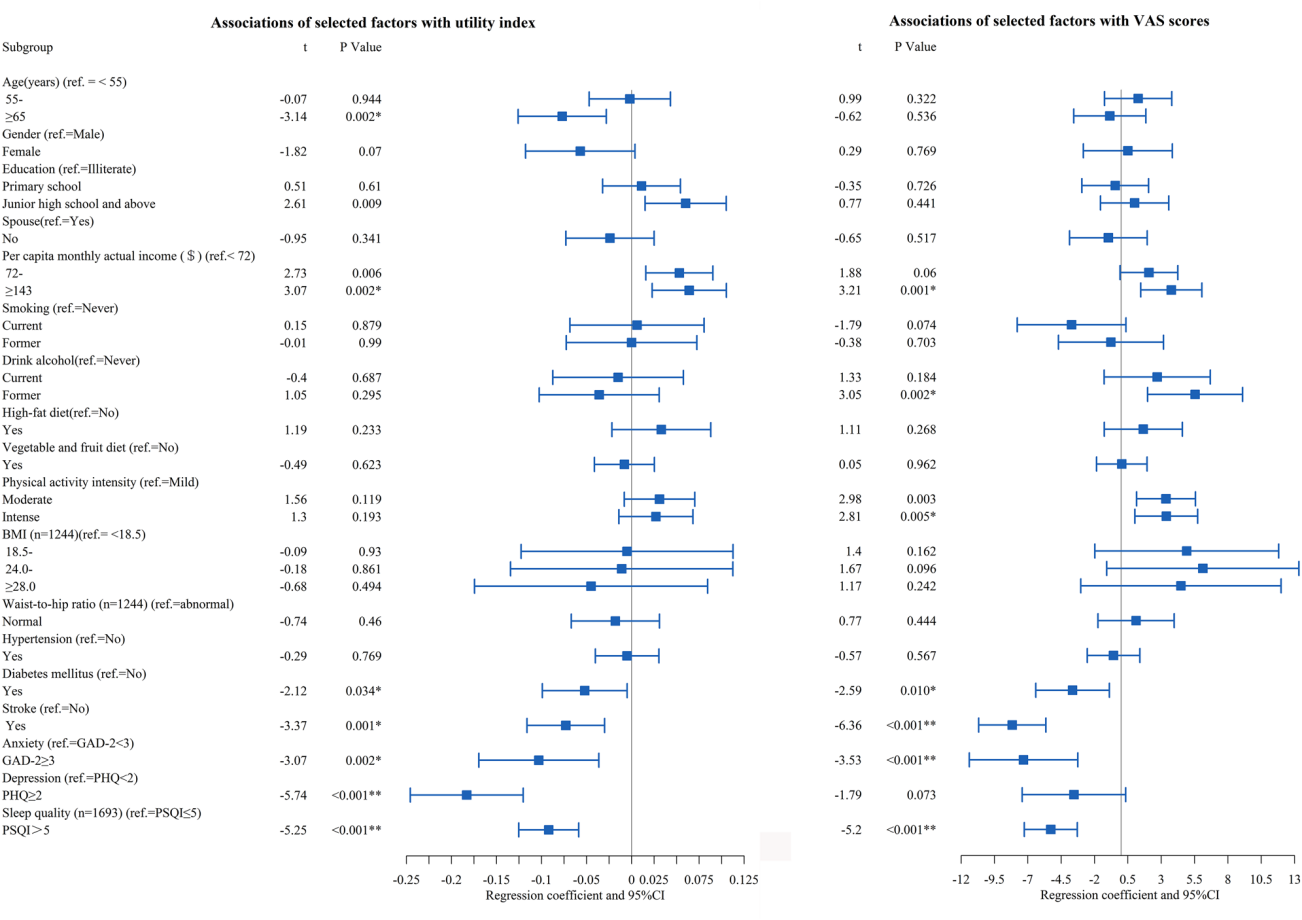
Factors associated with EQ-5D-5L utility index and Visual Analogue Scale scores in patients with coronary heart disease were analyzed by Tobit regression and Generalized linear model, respectively (n = 1247). The estimated effect and 95% confidence intervals were represented by black squares with the corresponding error bars. A two-sided *P* value 0.05 was considered statistically significant. *Notes*: GAD-2 Generalized Anxiety Disorder Scale-2, PHQ-2 Patient Health Questionnaire-2, PSQI the Pittsburgh Sleep Quality Index.

The correlation factors of the health utility index and VAS scores, as determined by the Tobit regression analyses and GLM, are shown in Fig. 2 and Table S2. CHD patients who were older (especially those aged > 65 years) had a lower utility index but did not have significantly different VAS scores. Participants who ever drank alcohol had lower VAS scores than those who never drank alcohol, but there were not a significant different in the utility index. CHD patients who performed intense physical activity had higher VAS scores than those who performed mild physical activity, but they did not have a significant difference in the utility index. CHD patients who had lower per capita monthly actual income, and patients with diabetes mellitus, stroke, had anxiety and poor sleep quality had significantly decreased health utility index and VAS scores.

## Discussion

Our study enriched the current literature about the relationship between the EQ-5D utility index, the domain-specific scores and the factors associated with each in a large sample of CHD patients in rural areas. The main findings were that the most frequently reported problems where in the PD dimension; CHD patients had lower per capita monthly actual income; and diabetes mellitus, stroke, had anxiety and poor sleep quality contributed to significantly lower health utility index and VAS scores. For the different dimensions of the EQ-5D, the influencing factors were varied.

The mean utility index and VAS score of patients with CHD were 0.914 and 69.44, respectively, which were lower than those of the urban Chinese population, with mean EQ-5D index and VAS scores of 0.957 and 86.0, respectively^29^, and lower than those of rural people with dyslipidaemia in Xinxiang, with mean EQ-5D index and VAS scores of 0.953 and 78.46, respectively^30^. This confirmed that CHD patients had a great impact on decreasing HRQoL in the Chinese rural population^31^. However, the EQ-5D index and EQ-VAS scores were higher than those CHD patients from Slovenia (0.60 and 58.6, respectively). The reason may be the different cross-walk value sets used^32^.

Our findings showed that 55.6% of CHD patients reported being in full health, which was higher than of the proportion of Australian adults reporting the same (42.8%)^33^. The possible reason may be that Chinese people in rural areas are more tolerant to diseases and they don’t take for granted that they are healthy^34^. However, problems in the PD domain were the most frequently reported (34.9%), and AD problems were the least frequently reported (12.5%) in CHD patients. CHD patients in 24 European countries had the most PD problems (58.9%), and SA problems were the least reported (11.6%)^10^. Different countries showed major differences in reported problems^10^. The reason may be the various cultures or the different cross-walk value sets used.

CHD patients had lower per capita monthly actual income, and a higher prevalence of diabetes mellitus, stroke, had anxiety and poor sleep quality, which significantly decreased the health utility index and VAS scores. Differences in income may be the reason that people living in urban places have a higher HRQoL than rural people^14^. CHD patients with higher income were less likely to have MO, SC, and UA problems. The Chinese government is dedicated to performing society wellness programmes and trying to improve the income and quality of life of national people^35^, and the situation will become increasingly better. CHD patients suffering from comorbidities such as diabetes and stroke were more likely to have a worse HRQoL^10^. Moreover, CHD patients with stroke had a higher risk of having problems with PD, and patients with hypertension reported more often suffering from problems with MO and UA. However, the findings did not agree with a study in which worse HRQoL in CHD patients was associated with comorbidities such as heart failure or peripheral artery disease but not stroke^36^. Pain is a common clinical problem in patients with stroke that not only increases depression and cognitive problems but also impairs quality of life^37^. CHD patients with stroke need more attention, especially those with PD problems.

Anxiety and depression play an important role in decreasing the HRQoL of CHD patients^38^. Although depression was not associated with the HRQoL of CHD patients in this study, CHD patients with depression had a higher risk of having problems with MO, SC, UA, PD, and AD. The reason may be that depression may indirectly affect HRQo^16^. In addition, previous research showed that rural elderly individuals had more depressive symptoms than urban elderly individuals^39^, and rural females had a higher prevalence of depressive disorders^40^. Therefore, depression screening and nonpharmacological intervention need to be performed, especially for rural female CHD patients.

CHD patients with poor sleep quality should be taken into consideration when planning rehabilitation, and sleep time is the one of the key influencing factors to increasing quality of life^41^. A study showed that adults with chronic disease who slept <7 or > 8 h were more likely to report poor or fair health and frequent mental distress, and a U-shaped relationship between sleep duration and HRQoL was found^42^. Meeting sleep guidelines (7–9 h/night) is associated with better HRQoL and reduced all-cause mortality risk in adults^43^. However, determining how to meet the sleep guidelines needs more study, and further research about how to improve sleep quality among CHD patients needs to be performed.

In addition, older CHD patients and those who were without spouses, had lower education, ever drank alcohol, had a high-fat diet, and had mild physical activity also need further attention. CHD patients who ever drank and had a high-fat diet took it for granted that they had a higher HRQoL. They did not realize the harm of drinking or a high-fat diet, so health education should be enhanced about the healthy lifestyles^44^.

This study reported the influencing factors of the EQ-5D utility index and its domain-specific scores in CHD patients based on a representative rural population in China, which guaranteed the reliability. The findings provide important implications. First, this study highlights the importance of the HRQoL of CHD patients in rural areas, which should encourage the government to focus more on the systematic management of CHD patients in rural areas. CHD patients with a low level of HRQoL need more assistance and resources. Second, the influencing factors of the EQ-5D utility index and the domain-specific scores in CHD patients provide insight into potential interventions to improve the HRQoL of CHD patients. Interventions focused on changing lifestyles and improving mental health and sleep quality may be designed by nurses, according to the diverse CHD patient populations and various settings, to improve HRQoL. However, several limitations in this study should be noted. First, CHD was self-reported by the participants, and the different types of CHD, severity of the disease, drug use, and other factors were not distinguished, which may have potentially biased our estimates of the impact on HRQoL in CHD patients. Second, this study was cross-sectional in design, so no causal relationships could be determined. Third, the EQ-5D-5L patient populations value set for China was developed from urban areas rather than rural areas, which may introduce bias to our study. Fourth, although a large sample in Henan was recruited in this study, the number of CHD patients in rural China is limited.

The EQ-5D utility index and VAS scores of rural CHD patients were lower than those of other rural populations. Pain/discomfort was the most frequently reported problem. Higher per capita monthly actual income, more physical activity, lower depression and higher sleep quality predict higher health utility index and VAS scores. Our national public health service should be involved in providing systematic management for CHD patients, especially regarding pain, depression, physical activity and sleep quality.

## Supplementary Information

Supplementary Information 1.

Supplementary Information 2.

## Data Availability

The datasets generated for this study are available on request to the corresponding author.
