# Supplementary material for: Health-related quality of life and its related factors in coronary heart disease patients: results from the Henan Rural Cohort study
Source: Sci Rep. 2021 Mar 3;11:5011. doi: 10.1038/s41598-021-84554-6 (PMC7930256; doi:10.1038/s41598-021-84554-6)
Supplement: Supplementary file 1 — Supplementary Information 1. [file 41598_2021_84554_MOESM1_ESM.doc]

**Health-related quality of life and its related factors in coronary heart disease patients: results from the Henan Rural Cohort study**

Yong-xia Mei^a,b,c^, Hui Wu^d,e,f^, Huan-yun Zhang^c^, Jian Hou^b^, Zhen-xiang Zhang^a^, Wei Liao^b^, Xiao-tian Liu^b^, Sheng-xiang Sang^b^, Zhen-xing Mao^b^, Dong-bin Yang^c^*, Chong-jian Wang^b^*,Wei-hong Zhang^a^*

^a^School of Nursing and Health, Zhengzhou University, Zhengzhou,China;

^b^Department of Epidemiology and Biostatistics, College of Public Health, Zhengzhou University, Zhengzhou, Henan, PR China.

^c^The people’s hospital of Hebi, 115 Jiuzhou Rd, Hebi,458000, Henan, China

^d^School of Public Health, Xinxiang Medical University, 601 Jinsui Rd, Xinxiang, 453000, Henan, China

^e^School of Public Health, Tianjin Medical University, 22 Qixiangtai Rd, Heping District, 300070 Tianjin, China

^f^Henan Province General Medical Educations and Research Center, Xinxiang, China

*Correspondence to: Dong-bin Yang, Chong-jian Wang and Wei-hong Zhang

Dong-bin Yang, PhD, Professor

Department of Neurosurgery of Hebi People's Hospital;

Hebi Neuroanatomical Laboratory, Hebi, 458030, China.

Email: [dongbinyang@126.com](mailto:dongbinyang@126.com)

Chong-jian Wang, PhD, Professor,

Department of Epidemiology and Biostatistics

College of Public Health, Zhengzhou University

100 Kexue Avenue, Zhengzhou, 450001, Henan, PR China.

1. mail: [tjwcj2005@126.com](mailto:tjwcj2005@126.com)

Wei-hong Zhang, PhD, Professor

School of Nursing and Health, Zhengzhou University, No. 100 Kexue Road, Zhengzhou, 450001, China. Email: [18638127788@163.com](mailto:18638127788@163.com)

Table S1 Risk factors for having problems in each EQ-5D dimension among CHD patients (n=1247): A multiple logistic regression analysis

| Subject characteristics | MO  OR (95%CI) | SC  OR (95%CI) | UA  OR (95%CI) | PD  OR (95%CI) | AD  OR (95%CI) |
| --- | --- | --- | --- | --- | --- |
| Age(years) |  |  |  |  |  |
| <55 | (ref) | (ref) | (ref) | (ref) | (ref) |
| 55~65 | 1.04 (0.67-1.63) | 1.24 (0.52-2.92) | 0.80 (0.44-1.48) | 0.97 (0.68-1.4) | 1.10 (0.62-1.93) |
| 65~ | 1.55 (0.99-2.44) | 2.30 (1.00-5.30) | 1.30 (0.71-2.37) | 1.63 (1.12-2.37)* | 1.25 (0.69-2.26) |
| Gender |  |  |  |  |  |
| Male | (ref) | (ref) | (ref) | (ref) | (ref) |
| Female | 1.27 (0.71-2.26) | 1.31 (0.45-3.78) | 1.53 (0.67-3.47) | 1.7 (1.05-2.73)* | 0.82 (0.42-1.62) |
| Education |  |  |  |  |  |
| illiterate | (ref) | (ref) | (ref) | (ref) | (ref) |
| Primary school | 1.04 (0.72-1.50) | 0.92 (0.55-1.56) | 0.85 (0.54-1.33) | 0.90 (0.65-1.25) | 1.17 (0.71-1.93) |
| Junior high school and above | 0.76 (0.51-1.14) | 0.37 (0.19-0.75)* | 0.53 (0.31-0.90)* | 0.66 (0.47-0.94)* | 1.04 (0.61-1.80) |
| Spouse |  |  |  |  |  |
| Yes | (ref) | (ref) | (ref) | (ref) | (ref) |
| No | 1.64 (1.10-2.47)* | 1.19 (0.65-2.19) | 2.07 (1.27-3.35)* | 1.16 (0.79-1.71) | 1.71 (1.00-2.92) |
| Per capita monthly actual income (＄) | |  |  |  | |
| <72 | (ref) | (ref) | (ref) | (ref) | (ref) |
| 72~143 | 0.56 (0.40-0.79) | 0.60 (0.36-1.01) | 1.02 (0.68-1.54) | 0.80 (0.6-1.08) | 0.92 (0.59-1.45) |
| 143~ | 0.54 (0.37-0.79) | 0.35 (0.17-0.69)* | 0.41 (0.23-0.71)* | 0.81 (0.59-1.11) | 0.74 (0.45-1.22) |
| Smoking status |  |  |  |  |  |
| Never | (ref) | (ref) | (ref) | (ref) | (ref) |
| Current | 1.20 (0.59-2.46) | 1.59 (0.46-5.52) | 1.34 (0.5-3.65) | 0.90 (0.5-1.63) | 0.44 (0.17-1.14) |
| Former | 1.66 (0.84-3.3) | 2.17 (0.65-7.22) | 1.56 (0.59-4.1) | 0.73 (0.41-1.32) | 0.44 (0.17-1.10) |
| Drinking status |  |  |  |  |  |
| Never | (ref) | (ref) | (ref) | (ref) | (ref) |
| Current | 1.07 (0.56-2.03) | 1.12 (0.42-3.01) | 1.96 (0.88-4.36) | 1.30 (0.73-2.31) | 0.73 (0.26-2.08) |
| Former | 0.44 (0.22-0.88)* | 0.37 (0.10-1.38) | 0.26 (0.07-0.92)* | 1.21 (0.71-2.05) | 1.09 (0.47-2.56) |
| High-fat diet |  |  |  |  |  |
| No |  |  |  |  | - |
| Yes | 0.50 (0.28-0.89)* | 0.74 (0.30-1.84) | 0.45 (0.19-1.04) | 0.97 (0.64-1.47) | 0.79 (0.40-1.57) |
| Physical activity intensity |  |  |  |  |  |
| Mild | (ref) | (ref) | (ref) | (ref) | (ref) |
| Moderate | 0.68 (0.48-0.96)* | 0.65 (0.38-1.11) | 0.60 (0.38-0.95)* | 1.25 (0.92-1.7) | 0.88 (0.56-1.40) |
| Intense | 0.72 (0.50-1.04) | 0.54 (0.30-0.97)* | 0.79 (0.49-1.25) | 1.19 (0.86-1.65) | 0.75 (0.46-1.24) |
| BMI (n=1244) |  |  |  |  |  |
| <18.5 | (ref) | (ref) | (ref) | (ref) | (ref) |
| 18.5≤BMI<24.0 | 0.97 (0.34-2.80) | 0.50 (0.13-1.94) | 1.23 (0.34-4.46) | 1.80 (0.69-4.72) | 0.93 (0.27-3.15) |
| 24.0≤BMI<28.0 | 0.92 (0.30-2.80) | 0.51 (0.12-2.25) | 0.96 (0.24-3.82) | 1.75 (0.64-4.8) | 0.86 (0.23-3.18) |
| ≥28.0 | 1.51 (0.47-4.83) | 0.70 (0.15-3.31) | 1.29 (0.31-5.4) | 2.57 (0.9-7.32) | 0.68 (0.17-2.72) |
| Centripetal obesity (n=1245) |  |  |  |  |  |
| No | (ref) | (ref) | (ref) | (ref) | (ref) |
| Yes | 1.12 (0.69-1.80) | 0.95 (0.45-1.99) | 1.04 (0.57-1.91) | 0.77 (0.52-1.14) | 1.07 (0.58-1.97) |
| Waist-to-hip ratio (n=1244) |  |  |  |  |  |
| Normal | (ref) | (ref) | (ref) | (ref) | (ref) |
| abnormal | 1.15 (0.72-1.84) | 1.58 (0.72-3.44) | 1.21 (0.66-2.21) | 1.14 (0.78-1.68) | 0.76 (0.43-1.36) |
| Hypertension |  |  |  |  |  |
| No | (ref) | (ref) | (ref) | (ref) | (ref) |
| Yes | 1.43 (1.05-1.95)* | 1.43 (0.89-2.29) | 1.47 (0.99-2.17) | 0.95 (0.73-1.25) | 1.24 (0.82-1.87) |
| Stroke |  |  |  |  |  |
| No | (ref) | (ref) | (ref) | (ref) | (ref) |
| Yes | 1.31 (0.91-1.9) | 1.37 (0.8-2.33) | 1.49 (0.95-2.34) | 1.35 (0.96-1.89) | 1.55 (0.97-2.48) |
| Anxiety (n=1246) |  |  |  |  |  |
| GAD-2<3 | (ref) | (ref) | (ref) | (ref) | (ref) |
| GAD-2≥3 | 0.77 (0.41-1.43) | 1.20 (0.52-2.74) | 0.71 (0.34-1.47) | 1.70 (1-2.91)* | 3.00 (1.63-5.51)* |
| Depression (n=1246) |  |  |  |  |  |
| PHQ<2 | (ref) | (ref) | (ref) | (ref) | (ref) |
| PHQ≥2 | 2.38 (1.36-4.18)* | 3.58 (1.69-7.59)* | 4.38 (2.31-8.3)* | 2.12 (1.28-3.52)* | 6.04 (3.45-10.57)** |
| Sleep quality (n=1229) |  |  |  |  |  |
| PSQI≤5 | (ref) | (ref) | (ref) | (ref) | (ref) |
| PSQI＞5 | 1.83 (1.35-2.48)* | 2.32 (1.44-3.72)* | 2.64 (1.79-3.88)* | 1.93 (1.48-2.51)* | 1.32 (0.88-1.97) |

CHD: Coronary Heart Disease

MO: Mobility; SC:Self-care; UA: Usual activities; PD: Pain/discomfort; AD: Anxiety/depression

GAD-2 Generalized Anxiety Disorder Scale-2, PHQ-2 Patient Health Questionnaire-2, PSQI the Pittsburgh Sleep Quality Index

* *P* <0.05; ** *P* <0.001
