# Supplementary material for: Health-related quality of life and its related factors in coronary heart disease patients: results from the Henan Rural Cohort study
Source: Sci Rep. 2021 Mar 3;11:5011. doi: 10.1038/s41598-021-84554-6 (PMC7930256; doi:10.1038/s41598-021-84554-6)
Supplement: Supplementary file 2 — Supplementary Information 2. [file 41598_2021_84554_MOESM2_ESM.doc]

**Health-related quality of life and its related factors in coronary heart disease patients: results from the Henan Rural Cohort study**

Yong-xia Mei^a,b,c^, Hui Wu^d,e,f^, Huan-yun Zhang^c^, Jian Hou^b^, Zhen-xiang Zhang^a^, Wei Liao^b^, Xiao-tian Liu^b^, Sheng-xiang Sang^b^, Zhen-xing Mao^b^, Dong-bin Yang^c^*, Chong-jian Wang^b^*,Wei-hong Zhang^a^*

^a^School of Nursing and Health, Zhengzhou University, Zhengzhou,China;

^b^Department of Epidemiology and Biostatistics, College of Public Health, Zhengzhou University, Zhengzhou, Henan, PR China.

^c^The people’s hospital of Hebi, 115 Jiuzhou Rd, Hebi,458000, Henan, China

^d^School of Public Health, Xinxiang Medical University, 601 Jinsui Rd, Xinxiang, 453000, Henan, China

^e^School of Public Health, Tianjin Medical University, 22 Qixiangtai Rd, Heping District, 300070 Tianjin, China

^f^Henan Province General Medical Educations and Research Center, Xinxiang, China

*Correspondence to: Dong-bin Yang, Chong-jian Wang and Wei-hong Zhang

Dong-bin Yang, PhD, Professor

Department of Neurosurgery of Hebi People's Hospital;

Hebi Neuroanatomical Laboratory, Hebi, 458030, China.

Email: [dongbinyang@126.com](mailto:dongbinyang@126.com)

Chong-jian Wang, PhD, Professor,

Department of Epidemiology and Biostatistics

College of Public Health, Zhengzhou University

100 Kexue Avenue, Zhengzhou, 450001, Henan, PR China.

1. mail: [tjwcj2005@126.com](mailto:tjwcj2005@126.com)

Wei-hong Zhang, PhD, Professor

School of Nursing and Health, Zhengzhou University, No. 100 Kexue Road, Zhengzhou, 450001, China. Email: [18638127788@163.com](mailto:18638127788@163.com)

Table S2 Tobit regression analyses and Generalized linear model on the health utility index and VAS scores

| Subject characteristics | Utility index | | | | VAS scores | | | |
| --- | --- | --- | --- | --- | --- | --- | --- | --- |
|  | COE | SE | *t* | *P* | COE | SE | *t* | *P* |
| Age(years) (ref.=<55) |  |  |  |  |  |  |  |  |
| 55~65 | -0.002 | 0.023 | -0.07 | 0.944 | 1.273 | 1.286 | 0.99 | 0.322 |
| 65~ | -0.077 | 0.025 | -3.14 | 0.002* | -0.853 | 1.379 | -0.62 | 0.536 |
| Gender (ref.=Male) |  |  |  |  |  |  |  |  |
| Female | -0.057 | 0.031 | -1.82 | 0.070 | 0.500 | 1.702 | 0.29 | 0.769 |
| Education (ref.=Illiterate) |  |  |  |  |  |  |  |  |
| Primary school | 0.011 | 0.022 | 0.51 | 0.610 | -0.448 | 1.277 | -0.35 | 0.726 |
| Junior high school and above | 0.060 | 0.023 | 2.61 | 0.009 | 1.007 | 1.308 | 0.77 | 0.441 |
| Spouse (ref.=Yes) |  |  |  |  |  |  |  |  |
| No | -0.024 | 0.025 | -0.95 | 0.341 | -0.961 | 1.484 | -0.65 | 0.517 |
| Per capita monthly actual income (＄) (ref.=<72) | |  |  |  |  |  |  |  |
| 72~143 | 0.053 | 0.019 | 2.73 | 0.006 | 2.081 | 1.105 | 1.88 | 0.060 |
| 143~ | 0.064 | 0.021 | 3.07 | 0.002* | 3.760 | 1.170 | 3.21 | 0.001* |
| Smoking status (ref.=Never) |  |  |  |  |  |  |  |  |
| Current | 0.006 | 0.038 | 0.15 | 0.879 | -3.720 | 2.080 | -1.79 | 0.074 |
| Former | 0.000 | 0.037 | -0.01 | 0.990 | -0.767 | 2.011 | -0.38 | 0.703 |
| Drinking status(ref.=Never) |  |  |  |  |  |  |  |  |
| Current | -0.015 | 0.037 | -0.40 | 0.687 | 2.698 | 2.030 | 1.33 | 0.184 |
| Former | -0.036 | 0.034 | 1.05 | 0.295 | 5.539 | 1.817 | 3.05 | 0.002* |
| High-fat diet (ref.=No) |  |  |  |  |  |  |  |  |
| Yes | 0.033 | 0.028 | 1.19 | 0.233 | 1.657 | 1.495 | 1.11 | 0.268 |
| Vegetable and fruit diet (ref.=No) |  |  |  |  |  |  |  |  |
| Yes | -0.008 | 0.017 | -0.49 | 0.623 | 0.046 | 0.965 | 0.05 | 0.962 |
| Physical activity intensity (ref.=Mild) |  |  |  |  |  |  |  |  |
| Moderate | 0.031 | 0.020 | 1.56 | 0.119 | 3.359 | 1.127 | 2.98 | 0.003 |
| Intense | 0.027 | 0.021 | 1.30 | 0.193 | 3.383 | 1.203 | 2.81 | 0.005* |
| BMI (n=1244)(ref.=<18.5) |  |  |  |  |  |  |  |  |
| 18.5≤BMI<24.0 | -0.005 | 0.060 | -0.09 | 0.930 | 4.915 | 3.518 | 1.40 | 0.162 |
| 24.0≤BMI<28.0 | -0.011 | 0.063 | -0.18 | 0.861 | 6.123 | 3.675 | 1.67 | 0.096 |
| ≥28.0 | -0.045 | 0.066 | -0.68 | 0.494 | 4.483 | 3.831 | 1.17 | 0.242 |
| Waist-to-hip ratio (n=1244) (ref.=abnormal) | |  |  |  |  |  |  |  |
| Normal | -0.018 | 0.025 | -0.74 | 0.460 | 1.115 | 1.456 | 0.77 | 0.444 |
| Hypertension (ref.=No) |  |  |  |  |  |  |  |  |
| Yes | -0.005 | 0.018 | -0.29 | 0.769 | -0.576 | 1.006 | -0.57 | 0.567 |
| Diabetes mellitus (ref.=No) |  |  |  |  |  |  |  |  |
| Yes | -0.052 | 0.024 | -2.12 | 0.034* | -3.646 | 1.407 | -2.59 | 0.010* |
| Stroke (ref.=No) |  |  |  |  |  |  |  |  |
| Yes | -0.073 | 0.022 | -3.37 | 0.001* | -8.166 | 1.285 | -6.36 | <0.001** |
| Anxiety (ref.=GAD-2<3) |  |  |  |  |  |  |  |  |
| GAD-2≥3 | -0.103 | 0.034 | -3.07 | 0.002* | -7.322 | 2.075 | -3.53 | <0.001** |
| Depression (ref.=PHQ<2) |  |  |  |  |  |  |  |  |
| PHQ≥2 | -0.183 | 0.032 | -5.74 | <0.001** | -3.542 | 1.978 | -1.79 | 0.073 |
| Sleep quality (n=1693) (ref.=PSQI≤5) |  |  |  |  |  |  |  |  |
| PSQI＞5 | -0.092 | 0.017 | -5.25 | <0.001** | -5.279 | 1.016 | -5.20 | <0.001** |

VAS: Visual Analogue Scale

GAD-2 Generalized Anxiety Disorder Scale-2, PHQ-2 Patient Health Questionnaire-2, PSQI the Pittsburgh Sleep Quality Index

* *P* <0.05; ** *P* <0.001
